# Seroprotection at Different Levels of the Healthcare System After Routine Vaccination With Diphtheria-Tetanus-Pertussis whole cell–Hepatitis B–*Haemophilus influenzae* Type B in Lao People’s Democratic Republic

**DOI:** 10.1093/cid/ciz143

**Published:** 2019-02-19

**Authors:** Lisa Hefele, Sengdavanh Syphan, Dalouny Xayavong, Anousin Homsana, Daria Kleine, Phetsavanh Chanthavilay, Phonethipsavanh Nouanthong, Kinnaly Xaydalasouk, Outavong Phathammavong, Somxay Billamay, Anonh Xeuatvongsa, Daniel Reinharz, Claude P Muller, Antony P Black

**Affiliations:** 1 Lao-Lux Laboratory, Institut Pasteur du Laos, Vientiane, Lao People’s Democratic Republic; 2 Department of Infection and Immunity, Luxembourg Institute of Health, Esch-sur-Alzette, Grand-Duchy of Luxembourg; 3 Institut de la Francophonie pour la Médecine Tropicale, Vientiane, Lao People’s Democratic Republic; 4 Luxembourg Development Cooperation Agency, Vientiane, Lao People’s Democratic Republic; 5 Children Hospital, Vientiane, Lao People’s Democratic Republic; 6 Expanded Programme on Immunisation, Vientiane, Lao People’s Democratic Republic; 7 Laboratoire national de santé, Dudelange, Grand-Duchy of Luxembourg

**Keywords:** immunogenicity, vaccination, hepatitis B, diphtheria, tetanus

## Abstract

**Background:**

The Lao People’s Democratic Republic continues to sustain a considerable burden of vaccine-preventable diseases because of incomplete vaccine coverage and weak vaccine responses. We have assessed seroconversion after routine vaccination with the pentavalent vaccine to capture weaknesses of vaccine management at the different levels of the healthcare system.

**Methods:**

A total of 1151 children (aged 8–28 months) with 3 documented doses of the pentavalent vaccine delivered at central hospitals in Vientiane and the provincial hospital, 3 district hospitals, and 10 health centers in Bolikhamxay province were enrolled. Sociodemographic information was collected with a standardized questionnaire. Serum samples were analyzed for antibodies against vaccine components, and bivariate and multivariable analyses were performed to identify risk factors for low vaccine responses.

**Results:**

Seroprotection rates at the provincial, district, and health center level were as high as in central hospitals, but seroprotection rates in areas covered by remote health centers were significantly lower. Protective levels also rapidly decreased with age at sampling. Seroprotection rates in Bolikhamxay against the different components reached 70%–77% and were up to 20% higher than in previous studies in the same region; 18.8% more children received the hepatitis B vaccine birth dose and the hepatitis B virus infection rate was 4 times lower.

**Conclusions:**

Vaccine immunogenicity has dramatically improved in a central province, likely due to training and investment in the cold chain. Nevertheless, there remains a need to focus on the “last mile” in remote areas were most children are vaccinated through outreach activities.

## Teaser

Seroprotection rates after vaccination with the pentavalent vaccine were assessed at different levels of the healthcare system in Laos. While seroprotection did not necessarily decline at lower levels of the healthcare system, they were particularly suboptimal in remote areas.

Despite improvements in healthcare in the Lao People’s Democratic Republic (PDR), the incidence of vaccine-preventable diseases remains high. Vaccination represents one of the most important public health interventions [1–3], but many children fail to respond to the pentavalent diphtheria-tetanus-pertussis (whole cell)–Hepatitis B–*Haemophilus influenzae* type B vaccine (DTPw-HepB-Hib) vaccine [4], which is scheduled at 6, 10, and 14 weeks after birth. A first dose against hepatitis B is given as a monovalent vaccine within 24 hours after birth (“birth dose”). Long-term protective titers against hepatitis B virus (HBV; ≥100 IU/L) were as low as 7.7% in children aged 8–28 months with 3 documented vaccinations in 2013/2014 [4]. Many reasons can account for vaccine failure. However, in the 2013/2014 study, the low levels of seroprotection could only be partially explained by rapid waning of antibodies, home birth, malnutrition, and potential geographic disparities. The sensitivity of vaccines to excessive heat or cold is a major challenge for immunization programs. Exposure of vaccines to freezing temperatures resulted in vaccines with impaired immunogenicity [5–8].

Interventions from the Ministry of Health (MoH), World Health Organization (WHO), UNICEF, the Luxembourg Development Agency (LuxDev), and others have prompted a thorough review of vaccine management within the healthcare system. In Lao PDR, healthcare is delivered at 4 levels. The central hospitals (CHs) in Vientiane represent the first level, followed by the provincial health offices, each supervising a provincial hospital (PH), and a number of district health offices and district hospitals (DHs). The health centers (HCs) that belong to the DHs represent the lowest level, providing basic medical and outreach services to the surrounding villages [9].

The distribution of vaccines follows the same administrative line. Upon arrival in the country, the vaccines are stored in a central storage facility in Vientiane, from where the facilities of the capital and the provincial levels are supplied. The provincial health office supervises distribution to the DHs from where connected HCs are supplied. Immunization services are offered either directly at the facilities or through outreach services [10, 11].

Considering previous weaknesses of the immunization program and recent interventions, we reassessed the immunogenicity of the pentavalent vaccine in Bolikhamxay. We included children with 3 documented vaccine doses. A particular focus of our study was the site/level within the healthcare system at which the vaccine was delivered in order to capture any systemic logistical issues. We also included children vaccinated at CHs in Vientiane that are a short distance from the central storage facilities, as a surrogate gold standard for other levels of the health system.

## METHODS

The study was approved by the Lao National Ethics Committee and by the internal ethics review board of the Institut Pasteur du Laos. The numbers of the ethical approval documents are (033/2017/NECHR, 032/2017/NECHR, 031/2017/NECHR, 056/2017/NECHR).

### Study Participants

Children were recruited from the Children’s Hospital, a CH in Vientiane and from the PH in Paksan; 3 DHs in Viengthong, Khamkheut, and Pakkading; and 10 HCs in Bolikhamxay (Supplementary Figure A1). All parents/guardians signed the informed consent form and could withdraw participation at any time. A standardized questionnaire was designed to collect information about the participant’s socioeconomic background, access to healthcare, history, and location of vaccination. Vaccination histories were verified in hospital records (HRs) at the healthcare facilities (HCFs) if available and/or on the vaccination cards.

At the Children’s Hospital, 319 children aged 8–23 months who had received all 3 doses of the pentavalent vaccine in a CH in Vientiane were enrolled between May 2017 and February 2018. Of 319 children, 209 (65.5%) were vaccinated with all 3 doses at the Children’s Hospital, 36 (11.3%) received 1 or 2 of their doses at another CH, and 74 (23.2%) received all 3 doses at another CH. We reviewed the vaccination cards for all participants. A digital version of the questionnaire was used to collect the information [12].

In Bolikhamxay Province, 819 children aged 8–28 months vaccinated with the pentavalent vaccine were recruited between March 2017 and July 2017. Each of the 3 injections was documented by the vaccination card (73.3%), the HR (83.2%), or both (56.4%). HCFs and villages were selected based on geographic location, population size, and travel time to the next highest-ranked facility. Participants recruited at a particular HCF were almost always also vaccinated there (93.2%), receiving all 3 doses either on site or through outreach. Nevertheless, some of the participants (6.8%) had been vaccinated for 1 or more of their doses at another HCF or by a mix. All data were double entered in EPIDATA version 3.1 [13].

### Serology

After informed consent, 5 mL of blood were collected by trained healthcare workers. Serum was separated by centrifugation on the day of collection and stored at 4°C for a maximum of 5 days and then at –20°C for a maximum of 2 months. Commercial enzyme-linked immunosorbent assay kits were used to determine antibodies against hepatitis B surface antigen (anti-HBs), hepatitis B core antigen (anti-HBc) (Diasorin), tetanus, and diphtheria (Euroimmun). Anti-HBc(+)/anti-HBs(–) sera were also tested for the presence of the hepatitis b surface antigen (HBsAg) (Diasorin). The results were compared to those for the sera from our 2013/2014 study, which were tested with the same kits in the same laboratory at the Institute Pasteur du Laos. In addition, samples collected in the present study were tested for antipertussis toxin (Euroimmun) and anti-Hib (Progen) in parallel with the sera from 2013/2014. Immunity was considered protective when participants had antidiphtheria titers ≥0.1 IU/mL, antitetanus titers >0.5 IU/mL, and anti-Hib titers >1.0 µg/mL. For the anti-HBs titers, 2 cutoffs were used: ≥10 IU/L and ≥100 IU/L (long-term protection). To date, there is no protective titer established for antibodies against *Bordetella pertussis*, which complicates the interpretation of these titers. Here, a titer of ≥22 U/mL was used as an indication of exposure either to the vaccine antigen or the pathogen [14], but the assay cannot differentiate between the two.

### Data Analyses

Data analyses were conducted using R software [15] with the following packages: tidyverse [16], pastecs [17], epitools [18], car [19], lmtest [20], MASS [21], and rcompanion [22]. As we were interested in the association between the place of vaccination and seroprotection in semiurban and rural areas, only those participants enrolled in Bolikhamxay who had received all doses of the vaccine at the same place were included in the regression analyses. In bivariate analyses, odds ratio, 95% confidence interval, and *P* value for seroprotected children were calculated. Only variables with *P* values < .2 were included in the multivariable analyses. A correlation value >0.5 or a variance inflation factor > 2–5 was considered as correlation. Since the variables “place of birth” and “district” correlated highly with the variables for place of vaccination, they were not included. From the variables that describe access to healthcare, we chose “travel time in rainy season” for inclusion in the regression analyses. The place of vaccination was grouped in different categories to assess seroprotection first on all 3 levels overall, then according to outreach services, and last according to remoteness (HCs grouped according to travel time to DH: <30 min, 30–60 min, >60 min). Binary regressions were performed using a stepwise method for removing variables not associated with the response variable, taking both the *P* value of the variable and the Akaike information criterion of the model into consideration.

## RESULTS

### Participant Characteristics

A total of 306 children aged 8–23 months were recruited from the CH. Nearly half of the mothers (48%) were aged >30 years and had completed secondary school (45.4%) (Supplementary Table A1). Typically, the child was accompanied by the mother (97.1%), lived <10 km from the nearest HCF (84.3%), was of Tai-Kadai ethnicity (97.7%), and was from a household with an income >2.000.000 Kip (81.4%), corresponding to approximately 232 USD.

A total of 819 children aged 8–28 months were recruited from Bolikhamxay. Typically, children were accompanied by their mothers (80.6%), most of whom were married (97.7%) and of Tai-Kadai ethnicity (84.4%). Most families lived <10 km away from the nearest HCF (72.2%). The characteristics of the participants depended strongly on the place of recruitment. More families were from ethnic minorities at the HC level, their monthly income was less, and more mothers had no education compared to the PH.

### Serological Profiles

In Vientiane, at the Children’s Hospital, nearly all children had detectable antibodies against diphtheria, tetanus, and Hib, but protective levels varied from 85.1% (tetanus) to 92.5% (diphtheria) and 99.3% (Hib; Table 1). Of participants, 86.9% had anti-HBs ≥10 IU/L, and 57.7% had anti-HBs ≥100 IU/L. Only 36.7% of the children had antipertussis levels ≥22 IU/L, indicating infection or vaccination.

**Table 1.** Overall Percentage of Protective and Detectable Antibody Levels Against Diphtheria, Tetanus, *Haemophilus influenzae* Type B, Pertussis, and Hepatitis B Virus at the Children’s Hospital in Vientiane (Aged 8–23 Months) and in Bolikhamxay Province (Aged 8–28 Months)

Antibody	Category	Titer Cutoff	Children’s Hospital		Bolikhamxay Province	
			(N = 303–305*)		(N = 809–817*)	
			n (%)	CI 95%	n (%)	CI 95%
Antidiphtheria	Protected	≥0.1 IU/mL	282 (92.5)	[89.5–95.4]	631 (77.3)	[74.5–80.2]
	Detectable	≥0.01 IU/mL	304 (99.7)	[99.0–100.3]	773 (94.7)	[93.2–96.3]
Antitetanus	Protected	>0.5 IU/mL	258 (85.1)	[81.1–89.2]	616 (75.3)	[72.4–78.3]
	Detectable	≥0.01 IU/mL	303 (100)	[100]	797 (97.4)	[96.4–98.5]
Anti–*Haemophilus influenzae* type B	Protected	>1 µg/mL	303 (99.3)	[98.4–100.3]	580 (71.7)	[68.6–74.8]
	Detectable	>0.1 µg/mL	305 (100)	[100]	805 (99.5)	[99.0–100]
Anti–hepatitis B surface antigen	Protected	≥10 IU/L	265 (86.9)	[83.1–90.7]	588 (72.0)	[68.9–75.1]
		≥100 IU/L	176 (57.7)	[52.2–63.2]	348 (42.6)	[39.2–46.0]
Antipertussis	Exposure	≥22 IU/mL	112 (36.7)	[31.3–42.1]	200 (24.6)	[21.6–27.6]
	Detectable	≥0.2 IU/mL	291 (95.4)	[93.1–97.8]	673 (82.8)	[80.2–85.4]

Abbreviation: CI, confidence interval.

^*^Due to sometimes low sample volume, not all samples could be tested.

**Figure 1.**
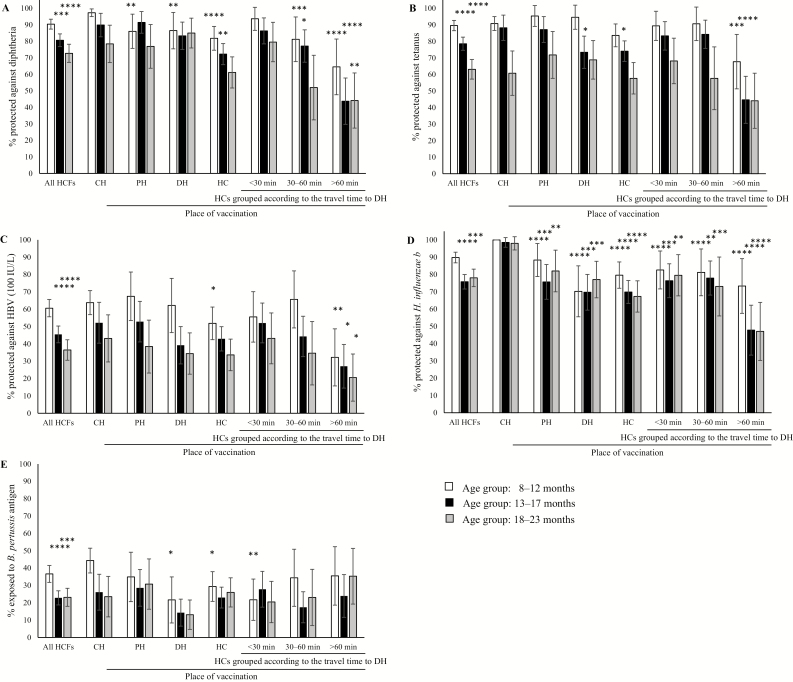
Seroprotection against diphtheria *(A)*, tetanus *(B)*, hepatitis B *(C)*, Hib *(D)*, and pertussis *(E)* according to place of vaccination in children aged 8–23 months grouped into 3 age ranges at the time of sample collection: 8–12 months, 13–17 months, and 18–23 months. Results are displayed with 95% confidence intervals. Only those children who were vaccinated with all 3 doses by the same immunization service either at a central hospital (CH) in Vientiane, the provincial hospital (PH), the district hospitals (DHs), or the health centers (HCs) were included in the graph. The HCs in the study in Bolikhamxay were further grouped according to travel time to the DH during the rainy season. Immunity was considered protective as described in methods. Seroprotection rates of children aged 13–17 months and 18–23 months at all healthcare facilities combined were compared to the youngest age group (8–12 months). Seroprotection rates between places of vaccination were always compared to the same age group at the CH. Abbreviations: *B. pertussis*, *Bordetella pertussis*; HCF, healthcare facility; HBV, hepatitis B virus; *H. influenzae* b, *Haemophilus influenzae* type b; *H. influenzae*, *Haemophilus influenzae*. *P ≤ .05, **P ≤ .01, ***P ≤ .001, ****P ≤ .0001.

In Bolikhamxay, protective levels of antibodies against tetanus, diphtheria, and Hib were lower by 9.8%, 15.1%, and 27.7%, respectively, compared to the CH. The 9%–28% differences between the children from Vientiane and Bolikhamxay disappeared when the age of the children was taken into account (Figure 1). In addition, seroprotection rates decreased significantly with the age or time since vaccination by 11.8% to 26.4%.

To compare the seroprotection at the different levels of the healthcare system, we age-matched the cohorts and selected only those participants who were vaccinated by the same immunization service with all 3 doses. Under these conditions, there was little difference between children vaccinated at the CH compared to those vaccinated at the PH, except for anti-Hib and the youngest age group for antidiphtheria. Furthermore, children who were vaccinated at HCs did not necessarily have a statistically significant lower overall antibody response than children vaccinated at higher-ranked facilities. However, large differences were observed when the 10 HCs were grouped according to their travel time to the respective DH (Figure 1).

### Serological Profiles in Bolikhamxay in 2017 and 2013/2014

**Figure 2.**
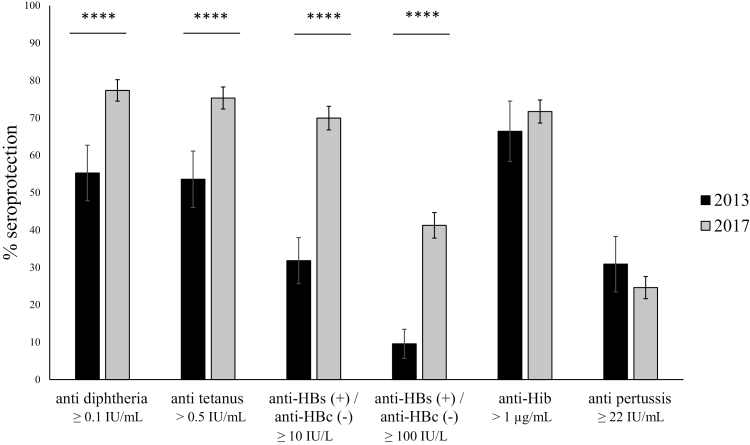
Comparison of the serological profiles of children aged 8–28 months enrolled in Bolikhamxay Province in 2017 and 2013/2014. Seroprotection rates are displayed with 95% confidence intervals. Immunity was considered protective as described in methods. Abbreviations: anti-HBs, anti–hepatitis B surface antigen; anti-HBc, anti–hepatitis B core antigen; anti-Hib, anti–*Haemophilus influenzae* type B. ****P ≤ .0001.

In 2017, seroprotection rates in Bolikhamxay seemed to have improved compared to 2013/2014 [4] (Figure 2). Seroprotection increased by more than 20% for antitetanus, antidiphtheria, and anti-HBs(+)/anti-HBc(–) (*P* < .0001), but only by 5% for anti-Hib (*P* = .22). However, 30.9% of the children had antibody levels ≥22 IU/mL against *B. pertussis* in 2013/2014 compared to only 24.9% in 2017. Similar improvements were found when only the villages in the Viengthong district were revisited (Supplementary Figure A2).

When antitetanus and antidiphtheria antibodies were used as a marker of vaccination with the pentavalent vaccine, 94% had detectable levels of antibodies against both tetanus and diphtheria compared to only 83.5% in 2013/2014 in the same age range. The proportion of children protected against tetanus, diphtheria, Hib, and HBV was more than twice as high in 2017 than in 2013/2014 (41.1% compared to 18.5%; Table 2).

**Table 2.** Relationship Between the Investigated Antibodies in Children From Bolikhamxay in 2017 and 2013/2014

Antibody	Protective Antibody Level			
	2017		2013/2014	
	n/N Tested	%	n/N Tested	%
Antitetanus, antidiphtheria, anti-HBs (≥10 IU/L)	414/812	51.0	27/162	16.7
Antitetanus, antidiphtheria, anti-HBs (≥100 IU/L)	270/812	33.3	15/162	9.3
Antitetanus, antidiphtheria, anti-HBs (≥10 IU/L), anti-Hib	331/806	41.1	23/124	18.5
Antitetanus, antidiphtheria, anti-HBs (≥100 IU/L), anti-Hib	227/806	28.1	13/124	10.5

Abbreviations: anti-HBs, antibodies against hepatitis b surface antigen; Hib, *Haemophilus influenzae* type B.

In 2017 compared to 2013/2014, the proportion of children who were anti-HBs positive (≥10 IU/L) and anti-HBc negative (Table 3) increased from 31.8% to 69.9% (*P* < .0001). Thus, the number of children who were protected after vaccination increased by more than 2-fold. At the same time, the number of children with an HBV infection decreased by 4-fold (13.18% to 3.11%, *P* < .0001) and the number of HBsAg carriers decreased by 2-fold (1.8% to 0.75%, *P* = .15). In 2017, 77% received the birth dose compared to only 58.2% in 2013/2014. In both years, children with the hepatitis B birth dose were more likely to be seropositive for anti-HBs(+)/anti-HBc(–); ≥10 IU/L (*P* = .01 in 2017 and 0.02 in 2013/2014) and less likely to be negative for both anti-HBs and anti-HBc (*P* = .01 in 2017 and 2013/2014). In 2017, 2.9% of the children who had received the birth dose were anti-HBc positive compared to 3.78% without the birth dose. In 2013/2014, those proportions were 14.8% and 10.9%, but in both years, the numbers of infections and of chronic carriers were too small to detect a significant impact of the birth dose.

**Table 3.** Hepatitis B Virus Serology of Children Tested for the Presence of the Hepatitis B Surface Antigen, Antibodies Against the Hepatitis B Surface Antigen and Antibodies Against the Hepatitis B Core Antigen

	2013/2014							2017						
			Hepatitis B Birth Dose				*P* Value^a^			Hepatitis B Birth Dose				*P* Value^a^
	All		No		Yes			All		No		Yes		
Hepatitis B Virus Serology	n	%	n	%	n	%		n	%	n	%	n	%	
Tested for anti-HBs^b^ and anti-HBc:	220	…	92	41.8^c^	128	58.2^ c^	…	805	…	185	23^ c^	620	77^ c^	…
Anti-HBs(+) and anti-HBc(–)	70	31.82	21	22.83	49	38.28	.02	563	69.94	114	61.62	449	72.42	.01
Anti-HBs(–) and anti-HBc(–)	121	55.00	61	66.30	60	46.88	.01	217	26.96	64	34.59	153	24.68	.01
Anti-HBc(+)	29	13.18	10	10.87	19	14.84	.63	25	3.11	7	3.78	18	2.90	.72
Anti-HBs(–), anti-HBc(+):														
HBsAg(–)	11	5.0	3	3.26	8	6.25	…	4	0.50	5	1.08	2	0.32	…
HBsAg(+)	4	1.82	2	2.17	2	1.56	.60^d^	6	0.75	2	1.08	4	0.65	1.00^d^

Abbreviations: anti-HBs, anti–hepatitis B surface antigen; anti-HBc, anti–hepatitis B core antigen; HBsAg, hepatitis B surface antigen.

^a^ *P* values for the association of the hepatitis B birth dose with serological profile.

^b^Cutoff used: ≥10 IU/L.

^c^Calculated to the total number of samples tested.

^d^Fisher test.

### Bivariate Analyses

Bivariate analyses were performed to identify factors associated with a *P* value less than 0.2 (Supplementary Table A2). Socioeconomic variables showed a strong tendency to correlate with each other due to ethnic and regional disparities. Therefore, in bivariate analyses (Supplementary Table A2), children were less likely to be seroprotected against all the vaccine antigens (except for pertussis) when they were of Hmong-Mien or Mon-Khmer ethnicity, aged >12 months, and vaccinated at remote HCs. In addition, low seroprotection against diphtheria, tetanus, and Hib was associated with poor education, long distance (>10 km or >40 min) to the HCF, or long-term breastfeeding and child age. Participants were more likely to be seroprotected when they lived in Paksan or when their family’s income exceeded 500.000 Kip per month. Receiving the hepatitis B birth dose was found to be only slightly associated with anti-HBs titers ≥100 IU/L (*P* < .2). Participants whose mothers stated to have received antenatal care services and more than 3 tetanus vaccination doses during their pregnancy had slightly higher chances to be protected against tetanus (*P* < .2).

### Multivariable Analyses

Logistic regression analyses were performed to identify risk factors for vaccine failure in Bolikhamxay (Table 4). In regression analysis, the age of the child and place of vaccination was found to be a strong predictor of seroprotection against tetanus, diphtheria, and HBV. Participants who were aged >12 months and vaccinated at remote HCs were less likely to be protected. In case of diphtheria, Hmong-Mien or Mon-Khmer ethnicity, living more than 10 km from the nearest HCF, and breastfeeding for longer than 6 months were additional risk factors. Participants were less likely to be seroprotected against Hib when they were aged >12 months and when they were of Hmong-Mien or Mon-Khmer ethnicity. The final model with the best fit for the Hib data also comprised the place of vaccination, although the variable was not statistically significant. None of the antipertussis regression models fit well to our data.

**Table 4.** Logistic Regression on Antidiphtheria Immunoglobulin G, Antitetanus, Anti–hepatitis B surface antigen, and Anti–*Haemophilus influenzae* Type B

			Factors Affecting Antidiphtheria IgG Level				Factors Affecting Antitetanus IgG Level				Factors Affecting Anti-HBs IgG Level				Factors affecting Anti–*Haemophilus influenzae* Type B IgG Level		
Variable	Category (N)^a^	% Protected	OR	95% CI	*P* Value	% Protected	OR	95% CI	*P* Value	% Protected	OR	95% CI	*P* Value	% Protected	OR	95% CI	*P* Value
Socioeconomic factors																	
Travel time to nearest healthcare facility (rainy season), min	<20 (428–431)	79.44	…	…	NS	77.73	….	…	NS	42.79	…	…	…	74.30	…	…	…
	20–40 (183–186)	82.26	…	…	…	78.92	…	…	…	45.41	…	…	…	71.04	…	…	…
	>40 (143–147)	65.99	…	…	…	63.70	…	…	…	41.50	…	…	…	69.23	…	…	…
Vaccinee-related factors																	
Ethnicity of parents/guardians	Tai-Kadai (619–624)	83.01	1.00	…	…	77.92	…	…	NS	44.55	…	…	NS	76.09	1.00	…	…
	Hmong-Mien + Mon-Khmer (135–138)	52.55	0.36	[0.22– 0.59]	**<.0001**	63.50	…	…	…	36.96	…	…	…	56.30	0.44	[0.28– 0.70]	**.0002**
Age of mother, y	≤ 20 (56–57)	80.36	…	…	…	77.19	…	…	…	35.09	1.00	…	…	76.79	…	…	…
	20–30 (467–471)	77.66	…	…	…	73.89	…	…	…	42.43	1.32	[0.74– 2.42]	.3535	73.12	…	…	…
	>30 (167–169)	76.19	…	…	…	79.17	…	…	…	50.89	1.90	[1.00– 3.69]	.0532	71.26	…	…	…
Age of participant, mo	≤12 (187–189)	83.60	1.00	…	…	88.36	1.00	…	…	57.22	1.00	…	…	79.68	1.00	…	…
	>12 (572–575)	75.52	0.50	[0.31– 0.79]	**.0040**	71.03	0.29	[0.17– 0.47]	**<.0001**	38.61	0.51	[0.35– 0.72]	**<.001**	70.19	0.55	[0.36– 0.82]	**.0049**
Place of birth	Home (141–144)	75.00	Removed due to correlation			76.92	Removed due to correlation			39.58	…	…	…	70.92	…	…	…
	Health center + district hospital (391–397)	74.87				72.47				44.08	…	…	…	71.36	…	…	…
	Provincial and central hospital + other (221–223)	83.86				79.37				43.89	…	…	…	75.68	…	…	…
Duration exclusive breastfeeding, mo	<6 (512–516)	82.91	1.00	…	…	69.92	…	…	NS	44.19	…	…	…	74.80	…	…	NS
	≥6 (242–246)	66.26	0.59	[0.40– 0.87]	**.0075**	77.91	…	…	…	41.06	…	…	…	67.77	…	…	…
Hepatitis B birth dose	Yes (586)	Not applicable				Not applicable				44.88	…	…	NS	Not applicable			
	No (176)									37.50	…	…	…				
Vaccine-related factors																	
District	Paksan (175)	82.86	Removed due to correlation			81.25	Removed due to correlation			45.71	…	…	…	74.86	Removed due to correlation		
	Khamkheut and Viengthong (420–428)	72.24				72.54				40.42	…	…	…	69.52			
	Pakkading (159–761)	77.53				75.78				47.80	…	…	…	77.99			
Place of vaccination with health centers grouped according to travel time to district hospital, min^b^	Provincial hospital (176)	82.95	1.00	…	…	81.25	1.00	…	…	45.45	1.00	…	…	74.43	1.00	…	…
	District hospital (175–178)	84.09	1.33	[0.75– 2.39]	.3296	75.44	0.74	[0.44– 1.24]	.255	42.13	0.95	[0.59– 1.52]	.832	72.00	0.97	[0.60– 1.57]	.9132
	<30 (166–168)	86.90	1.52	[0.83– 2.83]	.1793	80.84	0.93	[0.54– 1.62]	.802	50.00	1.26	[0.79– 2.00]	.3329	78.92	1.41	[0.84– 2.36]	.1939
	30 to 60 (126–128)	73.23	0.83	[0.46– 1.50]	.5324	80.47	0.94	[0.52– 1.70]	.833	47.66	1.15	[0.71– 1.88]	.5741	77.78	1.50	[0.86– 2.67]	.1574
	>60 (111–114)	50.00	0.41	[0.22– 0.76]	**.0050**	51.33	0.22	[0.13– 0.37]	**<.0001**	26.32	0.45	[0.26– 0.78]	**.0045**	54.95	0.65	[0.37– 1.17]	.1527

Data were dichotomized into participants with antidiphtheria IgG greater or less than 0.1 IU/mL, antitetanus IgG greater or less than 0.5 IU/mL, anti-HBs IgG greater or less than 100 IU/L, and anti-Hib IgG greater or less than 1.0 µg/mL. *P*-value <.05 highlighted in bold.

Abbreviations: CI, confidence interval; anti-HBs, anti–Hepatitis B surface antigen antibodies; IgG, immunoglobulin G; NS, not significant; OR, odds ratio.

^a^Total numbers for the categories vary between the analyses of the vaccine components; we could not list them individually due to space limitations.

^b^Each variable describing the place of vaccination was tested individually in the model; only the model with the best fit is reported here. Place of vaccination grouped according to the travel time from health center to the district hospital in the rainy season.

## DISCUSSION

Seroprotection levels against diphtheria, tetanus, Hib, and HBV (≥10 IU/L) reached 90%–100% for children aged <12 months who were vaccinated at CHs in Vientiane. These results are excellent after recent vaccination in the youngest age group and compare well with results in other countries [23]. The response to the pertussis antigen reached 44% in this setting.

When children were stratified by age, we found a considerable loss in seroprotection against diphtheria, tetanus, and HBV throughout the healthcare system. There was no similar consistent decay in antibodies against Hib and pertussis. The antibody levels at least against the pertussis antigen may also be explained by exposure to the pathogen. The apparent waning of the response to the different antigens may be due either to antibody waning with age or to gradual improvement in management of the cold chain. Overall improvements have been made since 2013 (P. Heimann, Luxembourg Development Cooperation, Laos, personal communication, oral communication, 15 March 2017 and 19 September 2018), but there are no data to quantitate this. In any case, the rapid loss of protection and apparent secondary vaccine failure require further attention.

Interestingly, seroprotection did not necessarily decline with a lower ranking of the facility within the healthcare system. While in absolute terms, seroprotection levels are relatively consistent from the provincial level to the HC level, on the HC level, the distance of the facilities to the respective DH was found to be a strong determinant of seroprotection against hepatitis B, diphtheria, and tetanus. The seroprotection rates are considerably lower in areas served by HCs located more than 60 minutes from its DH. In those remote HCs, 85% of the children who were vaccinated at more remote HCs were vaccinated during outreach sessions, whereas in the more proximate HCs, 68.3% to 72% were vaccinated by outreach. Most children of minorities are served by the more remote HCs, which explains their lower odds to be seroprotected against Hib and diphtheria. Thus, there is a need to specifically address issues that occur within the “last mile” of the vaccine cold chain, that is, the transport of vaccines from DH to HC and the transport from HC to the villages during outreach sessions. A recent study that assessed the cold chain in 2 provinces in Lao PDR reported exposure of vaccines to less than optimal temperatures at the district level [11].

The most encouraging result of our study is a dramatic improvement of seroprotection rates in 2017 compared to our earlier study in 2013/2014 in the same province [4]. This seems to be a reflection of the numerous interventions at the level of vaccine management, infrastructure, and training of staff throughout the cold chain during recent years, with the support and under the supervision of the MoH, LuxDev, UNICEF, the WHO country office and others. However, we cannot exclude that the shift in vaccine manufacturer from Berna/Crucell (Quinvaxem, now Janssen Vaccines Corp.) to the Serum Institute of India may be partially responsible for the enhanced immunogenicity, but we are not aware of similar observations from other countries.

The immune response to the hepatitis B component of the vaccine seemed to have increased by more than 30%. Nevertheless, about one third of the vaccinated children remain unprotected. In addition, about 29.3% of the children with an anti-HBs titer of 10–100 IU/L will require a booster dose within 3–6 months after vaccination. Currently, there is no additional booster program for hepatitis B in Lao PDR, leaving many children at risk, albeit at an age when the risk of a chronic infection is considerably lower. The improved response in 2017 was associated with a 4-fold decrease in HBV infection (13.2% in 2013/2014 to 3.1% in 2017).

In addition to geographic limitations, there were several others. Some of the positive serologies may be due to natural infections, at least in cases of diphtheria, *H. influenzae*, and *B. pertussis*. The specific place of vaccination (by outreach service or on site) was based on the parent’s recall as it could not be obtained through the HRs. We observed mismatches of vaccination dates in vaccination cards and HRs (manuscript in preparation). Although we can be confident that all children are vaccinated, some may have missed a dose despite documentation.

## CONCLUSIONS

Our data show a dramatic improvement in seroprotection rates after routine vaccination with the pentavalent vaccine after interventions to improve vaccine management. While seroconversion rates do not necessarily decline in peripheral facilities, the cold chain continues to be suboptimal particularly in remote areas, thus affecting marginal communities. In addition to strengthening vaccine management with a focus on the last mile of the supply chain, the introduction of a booster dose in remote settings should be considered.

## Supplementary Data

Supplementary materials are available at *Clinical Infectious Diseases* online. Consisting of data provided by the authors to benefit the reader, the posted materials are not copyedited and are the sole responsibility of the authors, so questions or comments should be addressed to the corresponding author.

ciz143_suppl_Supplementary_Materials

## Notes

***Acknowledgments.***The authors thank the participants, their families, and the healthcare staff for their participation and assistance. Furthermore, they thank LuxDev for providing support with the logistics throughout the sample collection and Dr Pauly for her advice regarding the data analyses.

***Financial support.***This work was supported by the Ministry of Foreign and European Affairs, Luxembourg (project “Lao Luxembourg Partnership for Research and Capacity Building in Infectious Disease Surveillance II”) and the Luxembourg Institute of Health and l’Agence universitaire de la Francophonie (AUF).

***Potential conflicts of interest.***All authors: No reported conflicts. All authors have submitted the ICMJE Form for Disclosure of Potential Conflicts of Interest. Conflicts that the editors consider relevant to the content of the manuscript have been disclosed.
