# Supplementary material for: Seroprotection at Different Levels of the Healthcare System After Routine Vaccination With Diphtheria-Tetanus-Pertussis whole cell–Hepatitis B–Haemophilus influenzae Type B in Lao People’s Democratic Republic
Source: Clin Infect Dis. 2019 Feb 19;69(12):2136–44. doi: 10.1093/cid/ciz143 (PMC6880335; doi:10.1093/cid/ciz143)

**Supplementary data**

**Tables**

**Appendix Table A1** Socio-demographic characteristics of participants and parents/guardians recruited at the Children’s Hospital in Vientiane Capital and at different health care facilities in Bolikhamxay province.

|  |  |  | **Children's Hospital** | |  | **Bolikhamxay** | |  | | |  | | | | |
| --- | --- | --- | --- | --- | --- | --- | --- | --- | --- | --- | --- | --- | --- | --- | --- |
|  |  |  | **(8 to 23 months)** | |  | **(8 to 28 months)** | |  | **Data from Bolikhamxay according to health care level** | | | | | | |
| **Variable** | **Categories** |  |  |  |  | **Total** | |  | **PH** | | | **DH** | | **HC** | |
|  |  |  | **N=306** | |  | **N = 819** | |  | **N = 191** | | | **N = 184** | | **N = 144** | |
|  |  |  | **n** | **%** |  | **n** | **%** |  | **n** | **%** | | **n** | **%** | **n** | **%** |
| Accompanying person | Mother |  | 297 | 97.1 |  | 660 | 80.6 |  | 158 | 82.7 | | 145 | 78.8 | 357 | 80.4 |
|  | Father |  | 6 | 2.0 |  | 57 | 7.0 |  | 12 | 6.3 | | 13 | 7.1 | 32 | 7.2 |
|  | Other |  | 3 | 1.0 |  | 102 | 12.5 |  | 21 | 11.0 | | 26 | 14.1 | 55 | 12.4 |
| Marital status of accompanying person | Unmarried |  | 1 | 0.3 |  | 2 | 0.2 |  | 0 | 0.0 | | 1 | 0.5 | 1 | 0.2 |
|  | Married |  | 305 | 99.7 |  | 791 | 96.6 |  | 189 | 99.0 | | 182 | 98.9 | 420 | 94.6 |
|  | Separated |  | 0 | 0.0 |  | 3 | 0.4 |  | 0 | 0.0 | | 1 | 0.5 | 2 | 0.5 |
|  | Divorced |  | 0 | 0.0 |  | 23 | 2.8 |  | 2 | 1.0 | | 0 | 0.0 | 21 | 4.7 |
|  | Not provided |  | 0 | 0.0 |  | 0 | 0.0 |  | 0 | 0.0 | | 0 | 0.0 | 0 | 0.0 |
| Age of the mother | < 20 years |  | 1 | 0.3 |  | 58 | 7.1 |  | 1 | 0.5 | | 13 | 7.1 | 44 | 9.9 |
|  | 20 – 30 years |  | 158 | 51.6 |  | 507 | 61.9 |  | 94 | 49.2 | | 120 | 65.2 | 293 | 66.0 |
|  | > 30 years |  | 147 | 48.0 |  | 185 | 22.6 |  | 70 | 36.6 | | 27 | 14.7 | 88 | 19.8 |
|  | NA |  |  |  |  | 69 | 8.4 |  | 26 | 13.6 | | 24 | 13.0 | 19 | 4.3 |
| Distance to nearest health care facility | < 10 km |  | 258 | 84.3 |  | 591 | 72.2 |  | 140 | 73.3 | | 170 | 92.4 | 281 | 63.3 |
|  | > 10 km |  | 48 | 15.7 |  | 228 | 27.8 |  | 51 | 26.7 | | 14 | 7.6 | 163 | 36.7 |
| Travel time to HCF (dry season) | < 20 min |  | 211 | 69.0 |  | 554 | 67.6 |  | 140 | 73.3 | | 172 | 93.5 | 242 | 54.5 |
|  | 20 - 40 min |  | 88 | 28.8 |  | 214 | 26.1 |  | 51 | 26.7 | | 12 | 6.5 | 151 | 34.0 |
|  | > 40 min |  | 7 | 2.3 |  | 51 | 6.2 |  | 0 | 0.0 | | 0 | 0.0 | 51 | 11.5 |
| Travel time to HCF (rainy season) | < 20 min |  | 196 | 64.1 |  | 465 | 56.8 |  | 101 | 52.9 | | 157 | 85.3 | 207 | 46.6 |
|  | 20 - 40 min |  | 100 | 32.7 |  | 199 | 24.3 |  | 90 | 47.1 | | 15 | 8.2 | 94 | 21.2 |
|  | > 40 min |  | 10 | 3.3 |  | 155 | 18.9 |  | 0 | 0.0 | | 12 | 6.5 | 143 | 32.2 |
| Mode of transport to HCF | Private car or private Tuktuk |  | 230 | 75.2 |  | 117 | 14.3 |  | 71 | 37.2 | | 37 | 20.1 | 9 | 2.0 |
|  | Other peopls's car |  | 0 | 0.0 |  | 9 | 1.1 |  | 0 | 0.0 | | 2 | 1.1 | 7 | 1.6 |
|  | Bus or Tuktuk |  | 1 | 0.3 |  | 6 | 0.7 |  | 0 | 0.0 | | 5 | 2.7 | 1 | 0.2 |
|  | by foot |  | 1 | 0.3 |  | 34 | 4.2 |  | 4 | 2.1 | | 2 | 1.1 | 28 | 6.3 |
|  | Motorcycle |  | 74 | 24.2 |  | 652 | 79.6 |  | 116 | 60.7 | | 138 | 75.0 | 398 | 89.6 |
|  | Partic,/Parents never go there (…) |  | 0 | 0.0 |  | 0 | 0.0 |  | 0 | 0.0 | | 0 | 0.0 | 0 | 0.0 |
|  | Other |  | 0 | 0.0 |  | 1 | 0.1 |  | 0 | 0.0 | | 0 | 0.0 | 1 | 0.2 |
| Ethnicity of parents / guardian | Mon-Khmer |  | 0 | 0.0 |  | 36 | 4.4 |  | 1 | 0.5 | | 7 | 3.8 | 28 | 6.3 |
|  | Tai-Kadai |  | 299 | 97.7 |  | 676 | 82.5 |  | 190 | 99.5 | | 165 | 89.7 | 321 | 72.3 |
|  | Hmong-Mien |  | 3 | 1.0 |  | 107 | 13.1 |  | 0 | 0.0 | | 12 | 6.5 | 95 | 21.4 |
|  | Other |  | 4 | 1.3 |  |  |  |  |  |  | |  |  |  |  |
| Occupation of mother | Housewife |  | 83 | 27.1 |  | 239 | 29.2 |  | 89 | 46.6 | | 69 | 37.5 | 81 | 18.2 |
|  | Labourer |  | 4 | 1.3 |  | 14 | 1.7 |  | 6 | 3.1 | | 4 | 2.2 | 4 | 0.9 |
|  | Farmer |  | 0 | 0.0 |  | 389 | 47.5 |  | 24 | 12.6 | | 59 | 32.1 | 306 | 68.9 |
|  | Trader |  | 76 | 24.8 |  | 66 | 8.1 |  | 22 | 11.5 | | 17 | 9.2 | 27 | 6.1 |
|  | Gov. employee |  | 120 | 39.2 |  | 106 | 12.9 |  | 49 | 25.7 | | 32 | 17.4 | 25 | 5.6 |
|  | Priv. employee |  | 22 | 7.2 |  | 4 | 0.5 |  | 1 | 0.5 | | 2 | 1.1 | 1 | 0.2 |
|  | Other |  | 1 | 0.3 |  | 1 | 0.1 |  | 0 | 0.0 | | 1 | 0.5 | 0 | 0.0 |
| Monthly income of household | < 500,000 Kip |  | 0 | 0.0 |  | 128 | 15.6 |  | 12 | 6.3 | | 12 | 6.5 | 104 | 23.4 |
|  | 500,000 – 1,000,000 Kip |  | 3 | 1.0 |  | 211 | 25.8 |  | 20 | 10.5 | | 50 | 27.2 | 141 | 31.8 |
|  | 1,000,000 – 2,000,000 Kip |  | 49 | 16.0 |  | 336 | 41.0 |  | 108 | 56.5 | | 80 | 43.5 | 148 | 33.3 |
|  | > 2,000,000 Kip |  | 249 | 81.4 |  | 139 | 17.0 |  | 50 | 26.2 | | 42 | 22.8 | 47 | 10.6 |
|  | Not provided / unknown |  | 5 | 1.6 |  | 5 | 0.6 |  | 1 | 0.5 | | 0 | 0.0 | 4 | 0.9 |
| Completed level of education of mother | None |  | 0 | 0.0 |  | 70 | 8.5 |  | 3 | 1.6 | | 12 | 6.5 | 55 | 12.4 |
|  | Primary School |  | 7 | 2.3 |  | 330 | 40.3 |  | 40 | 20.9 | | 55 | 29.9 | 235 | 52.9 |
|  | Secondary School |  | 139 | 45.4 |  | 292 | 35.7 |  | 73 | 38.2 | | 83 | 45.1 | 136 | 30.6 |
|  | Prof. training / College |  | 82 | 26.8 |  | 75 | 9.2 |  | 41 | 21.5 | | 22 | 12.0 | 12 | 2.7 |
|  | University |  | 78 | 25.5 |  | 52 | 6.3 |  | 34 | 17.8 | | 12 | 6.5 | 6 | 1.4 |
| Receive Antenatal Care | No |  | 1 | 0.3 |  | 72 | 8.8 |  | 2 | 1.0 | | 16 | 8.7 | 54 | 12.2 |
|  | Yes |  | 304 | 99.3 |  | 742 | 90.6 |  | 187 | 97.9 | | 167 | 90.8 | 388 | 87.4 |
|  | Unknown |  | 1 | 0.3 |  | 5 | 0.6 |  | 2 | 1.0 | | 1 | 0.5 | 2 | 0.5 |
| Age of child | ≤ 12 months |  | 186 | 60.8 |  | 201 | 24.5 |  | 46 | 24.1 | | 41 | 22.3 | 114 | 25.7 |
|  | > 12 months |  | 120 | 39.2 |  | 618 | 75.5 |  | 145 | 75.9 | | 143 | 77.7 | 330 | 74.3 |
| Gender of the child | Male |  | 156 | 51.0 |  | 433 | 52.9 |  | 91 | 47.6 | | 90 | 48.9 | 252 | 56.8 |
|  | Female |  | 150 | 49.0 |  | 386 | 47.1 |  | 100 | 52.4 | | 94 | 51.1 | 192 | 43.2 |
| Place of birth | Home |  | 0 | 0.0 |  | 149 | 18.2 |  | 7 | 3.7 | | 25 | 13.6 | 117 | 26.4 |
|  | Health center |  | 0 | 0.0 |  | 167 | 20.4 |  | 0 | 0.0 | | 1 | 0.5 | 166 | 37.4 |
|  | District hospital |  | 0 | 0.0 |  | 259 | 31.6 |  | 3 | 1.6 | | 134 | 72.8 | 122 | 27.5 |
|  | Provincial hospital |  | 8 | 2.6 |  | 201 | 24.5 |  | 158 | 82.7 | | 16 | 8.7 | 27 | 6.1 |
|  | Central hospital |  | 298 | 97.4 |  | 24 | 2.9 |  | 11 | 5.8 | | 7 | 3.8 | 6 | 1.4 |
|  | Other |  | 0 | 0.0 |  | 19 | 2.3 |  | 12 | 6.3 | | 1 | 0.5 | 6 | 1.4 |
| Exclusive breast-feeding | Yes |  | 270 | 88.2 |  | 72 | 8.8 |  | 167 | 87.4 | | 159 | 86.4 | 421 | 94.8 |
|  | No |  | 36 | 11.8 |  | 747 | 91.2 |  | 24 | 12.6 | | 25 | 13.6 | 23 | 5.2 |
|  |  |  |  |  |  |  |  |  |  |  | |  |  |  |  |
| Duration exclusive breast-feeding | < 6 months |  | 130 | 42.5 |  | 552 | 67.4 |  | 148 | 77.5 | | 118 | 64.1 | 286 | 64.4 |
|  | ≥ 6 months |  | 176 | 57.5 |  | 267 | 32.6 |  | 43 | 22.5 | | 66 | 35.9 | 158 | 35.6 |
| Age of addition of rice to diet | < 6 months |  | 8 | 2.6 |  | 309 | 37.7 |  | 80 | 41.9 | | 68 | 37.0 | 161 | 36.3 |
|  | ≥ 6 months |  | 298 | 97.4 |  | 510 | 62.3 |  | 111 | 58.1 | | 116 | 63.0 | 283 | 63.7 |
| Number of siblings | < 2 |  | 153 | 50.0 |  | 266 | 32.5 |  | 64 | 33.5 | | 61 | 33.2 | 141 | 31.8 |
|  | ≥ 2 |  | 153 | 50.0 |  | 553 | 67.5 |  | 127 | 66.5 | | 123 | 66.8 | 303 | 68.2 |
| Number of household members | < 6 |  | 300 | 98.0 |  | 432 | 52.7 |  | 123 | 64.4 | | 97 | 52.7 | 212 | 47.7 |
|  | ≥ 6 |  | 6 | 2.0 |  | 387 | 47.3 |  | 68 | 35.6 | | 87 | 47.3 | 232 | 52.3 |

**Appendix Table A2** Bivariate analyses: Data were dichotomized into participants with anti-Diphtheria IgG greater or less than 0.1 IU/ml, anti-tetanus IgG greater or less than 0.5 IU/ml, anti-HBs IgG greater or less than 100 IU/L, anti-Hib IgG greater or less than 1.0 µg/ml and anti-Pertussis greater or less than 22 IU/ml.

|  |  | **anti-diphtheria** | | | | **anti-tetanus** | | | | **anti-HBs** | | | | **anti-Hib** | | | | **anti-pertussis** | | | |
| --- | --- | --- | --- | --- | --- | --- | --- | --- | --- | --- | --- | --- | --- | --- | --- | --- | --- | --- | --- | --- | --- |
| **Variable** | **Categories (N)^1^** | **% protected** | **OR** | **95% CI** | **p-value** | **% protected** | **OR** | **95% CI** | **p-value** | **% protected** | **OR** | **95% CI** | **p-value** | **% protected** | **OR** | **95% CI** | **p-value** | **% protected** | **OR** | **95% CI** | **p-value** |
| **Socio-economic factors** | |  |  |  |  |  |  |  |  |  |  |  |  |  |  |  |  |  |  |  |  |
| **Occupation of mother** | Housewife (219-220) | 85.39 | 1.00 |  |  | 80.00 | 1.00 |  |  | 48.64 | 1.00 |  |  | 74.43 |  |  | NS | 27.40 |  |  | NS |
|  | Labourer + Farmer (374-378) | 71.16 | 0.42 | [0.27-0.65] | **<0.0001** | 71.43 | 0.63 | [0.42-0.93] | **0.0249** | 41.16 | 0.74 | [0.53-1.03] | **0.0876** | 69.79 |  |  |  | 23.20 |  |  |  |
|  | Trader + Employee + Other (161-163) | 81.71 | 0.76 | [0.44-1.32] | 0.4004 | 78.05 | 0.89 | [0.54-1.46] | 0.7035 | 40.49 | 0.72 | [0.48-1.08] | **0.1203** | 76.40 |  |  |  | 24.54 |  |  |  |
| **Level of education of mother** | None (68) | 66.18 | 1.00 |  |  | 61.76 | 1.00 |  |  | 41.18 |  |  | NS | 57.35 | 1.00 |  |  | 20.59 |  |  | NS |
|  | Primary school (300-306) | 77.56 | 1.77 | [1.00-3.12] | **0.0607** | 75.49 | 1.91 | [1.10-3.32] | **0.0241** | 46.56 |  |  |  | 71.33 | 1.85 | [1.08-3.18] | **0.0297** | 24.83 |  |  |  |
|  | Secondary school + University (386-390) | 79.49 | 1.98 | [1.13-3.46] | **0.0185** | 77.58 | 2.14 | [1.24-3.69] | **0.009** | 40.87 |  |  |  | 76.17 | 2.38 | [1.39-4.06] | **0.0026** | 25.32 |  |  |  |
| **Household income** | < 500,000 Kip (121-125) | 65.85 | 1.00 |  |  | 65.85 | 1.00 |  |  | 40.80 |  |  | NS | 63.64 | 1.00 |  |  | 28.69 |  |  | NS |
|  | 500,000 -1,000,000 Kip (199) | 83.42 | 2.61 | [1.54-4.42] | **0.0004** | 77.39 | 1.77 | [1.08-2.92] | **0.0281** | 45.23 |  |  |  | 71.86 | 1.46 | [0.90-2.36] | **0.1365** | 23.12 |  |  |  |
|  | > 1,000,000 Kip (434-440) | 78.13 | 1.85 | [1.20-2.87] | **0.0064** | 77.05 | 1.74 | [1.13-2.69] | **0.0139** | 42.92 |  |  |  | 75.35 | 1.75 | [1.14-2.68] | **0.0149** | 24.31 |  |  |  |
| **Travel time to nearest HCF (rainy season)** | < 20 min (428-431) | 79.44 | 1.00 |  |  | 77.73 | 1.00 |  |  | 42.79 |  |  | NS | 74.30 |  |  | NS | 23.44 |  |  | NS |
|  | 20 - 40 min (183-186) | 82.26 | 1.20 | [0.77-1.87] | 0.4418 | 78.92 | 1.07 | [0.70-1.63] | 0.8318 | 45.41 |  |  |  | 71.04 |  |  |  | 27.92 |  |  |  |
|  | > 40 min (143-147) | 65.99 | 0.50 | [0.33-0.76] | **0.0016** | 63.70 | 0.50 | [0.33-0.76] | **0.0014** | 41.50 |  |  |  | 69.23 |  |  |  | 25.00 |  |  |  |
| **Travel time to nearest HCF (dry season)** | < 20 min (510-513) | 79.84 | 1.00 |  |  | 77.58 | 1.00 |  |  | 43.66 |  |  | NS | 74.31 | 1.00 |  |  | 24.19 |  |  | NS |
|  | 20 - 40 min (197-200) | 78.50 | 0.92 | [0.62-1.38] | **0.6810** | 76.50 | 0.94 | [0.64-1.39] | 0.7659 | 44.22 |  |  |  | 72.59 | 0.92 | [0.63-1.33] | 0.6346 | 24.59 |  |  |  |
|  | > 40 min (49-50) | 50.00 | 0.25 | [0.14-0.46] | **<0.0000** | 46.94 | 0.26 | [0.14-0.46] | **<0.0000** | 34.00 |  |  |  | 53.19 | 0.39 | [0.21-0.72] | **0.0033** | 26.39 |  |  |  |
| **Distance to nearest HCF** | < 10 km (543-547) | 78.90 | 1.00 |  |  | 76.78 | 1.00 |  |  | 43.69 |  |  | NS | 74.77 | 1.00 |  |  | 25.32 |  |  | NS |
|  | >10 km (211-216) | 74.07 | 0.76 | [0.53-1.10] | **0.1501** | 71.63 | 0.76 | [0.53-1.09] | **0.1612** | 41.86 |  |  |  | 66.82 | 0.68 | [0.48-0.96] | **0.0296** | 23.11 |  |  |  |
| **Vaccinee related factors** | |  |  |  |  |  |  |  |  |  |  |  |  |  |  |  |  |  |  |  |  |
| **Ethnicity of parents / guardians** | Tai-Kadai (619-624) | 83.01 | 1.00 |  |  | 77.92 | 1.00 |  |  | 44.55 | 1.00 |  |  | 76.09 | 1.00 |  |  | 25.65 |  |  | NS |
|  | Hmong-Mien + Mon-Khmer (135-138) | 52.55 | 0.23 | [0.15-0.34] | **<0.0001** | 63.50 | 0.49 | [0.33-0.73] | **0.0006** | 36.96 | 0.73 | [0.50-1.07] | **0.1074** | 56.30 | 0.40 | [0.27-0.60] | **<0.0001** | 20.44 |  |  |  |
| **Age of participant** | ≤ 12 months (187-189) | 83.60 | 1.00 |  |  | 88.36 | 1.00 |  |  | 57.22 | 1.00 |  |  | 79.68 | 1.00 |  |  | 29.26 | 1.00 |  |  |
|  | >12 months (572-575) | 75.52 | 0.61 | [0.39-0.93] | **0.0209** | 71.03 | 0.32 | [0.20-0.52] | **<0.0001** | 38.61 | 0.47 | [0.34-0.66] | **<0.0001** | 70.19 | 0.60 | [0.40-0.90] | **0.0138** | 23.20 | 0.73 | [0.50-1.06] | **0.0982** |
| **Place of birth** | Home (141-144) | 75.00 | 1.00 |  |  | 76.92 | 1.00 |  | NS | 39.58 |  |  | NS | 70.92 |  |  | NS | 23.78 | 1.00 |  |  |
|  | Health center + District hospital (391-397) | 74.87 | 0.99 | [0.64-1.54] | 1.0000 | 72.47 | 0.79 |  |  | 44.08 |  |  |  | 71.36 |  |  |  | 21.94 | 0.90 | [0.57-1.42] | 0.6414 |
|  | Provincial & Central hospital + Other (221-223) | 83.86 | 1.73 | [1.03-2.91] | **0.0435** | 79.37 | 0.87 |  |  | 43.89 |  |  |  | 75.68 |  |  |  | 30.18 | 1.39 | [0.86-2.24] | **0.1898** |
| **Duration exclusive breastfeeding** | < 6 months (512-516) | 82.91 | 1.00 |  |  | 77.91 | 1.00 |  |  | 44.19 |  |  | NS | 74.80 | 1.00 |  |  | 23.05 |  |  | **NS** |
|  | ≥ 6 months (242-246) | 66.26 | 0.40 | [0.29-0.57] | **<0.0001** | 69.92 | 0.66 | [0.47-0.93] | **0.0194** | 41.06 |  |  |  | 67.77 | 0.71 | [0.51-0.99] | **0.0449** | 25.49 |  |  |  |
| **Vaccine related factors** | |  |  |  |  |  |  |  |  |  |  |  |  |  |  |  |  |  |  |  |  |
| **District** | Paksan (175) | 82.86 | 1.00 |  |  | 81.25 | 1.00 |  |  | 45.71 |  |  | NS | 74.86 | 1.00 |  |  | 32.00 | 1.00 |  |  |
|  | Khamkheut & Viengthong (420-428) | 72.24 | 0.54 | [0.34-0.84] | **0.0066** | 72.54 | 0.59 | [0.38-0.92] | **0.0219** | 40.42 |  |  |  | 69.52 | 0.77 | [0.51-1.14] | **0.1987** | 22.93 | 0.63 | [0.43-0.93] | **0.0235** |
|  | Pakkading (159-761) | 77.53 | 1.24 | [0.69-2.24] | 0.5496 | 75.78 | 0.70 | [0.41-1.18] | 0.2285 | 47.80 |  |  |  | 77.99 | 1.19 | [0.72-1.98] | 0.5216 | 21.38 | 0.58 | [0.35-0.95] | **0.0356** |
| **Location of vaccination: Groups** | |  |  |  |  |  |  |  |  |  |  |  |  |  |  |  |  |  |  |  |  |
| **All levels** | Provincial hospital (176) | 82.95 | 1.00 |  |  | 81.25 | 1.00 |  |  | 45.45 |  |  | NS | 74.43 |  |  | NS | 31.82 | 1.00 |  |  |
|  | District hospital 8176-178) | 84.09 | 1.08 | [0.62-1.91] | 0.8859 | 75.54 | 0.72 | [0.43-1.21] | 0.2446 | 42.13 |  |  |  | 72.00 |  |  |  | 15.34 | 0.39 | [0.23-0.65] | **0.0004** |
|  | Health center (403-409) | 72.37 | 0.54 | [0.34-0.84] | **0.0063** | 72.55 | 0.61 | [0.39-0.94] | **0.0283** | 42.65 |  |  |  | 71.96 |  |  |  | 25.68 | 0.74 | [0.50-1.09] | **0.1309** |
| **All levels divided in outreach service and vaccination at facility** | Provincial hospital (176) | 82.95 | 1.00 |  |  | 81.25 |  |  | NS | 45.45 |  |  | NS | 74.43 | 1.00 |  |  | 31.82 | 1.00 |  |  |
|  | District hospital – at facility (113-114) | 80.70 | 0.86 | [0.47-1.58] | 0.6409 | 75.44 |  |  |  | 42.98 |  |  |  | 78.76 | 1.27 | [0.72-2.24] | 0.4799 | 16.81 | 0.43 | [0.24-0.78] | **0.0057** |
|  | District hospital – by outreach service (62-64) | 90.32 | 1.92 | [0.76-4.86] | 0.2166 | 76.56 |  |  |  | 40.63 |  |  |  | 59.68 | 0.51 | [0.28-0.94] | **0.0351** | 12.70 | 0.31 | [0.14-0.70] | **0.0028** |
|  | Health center – at facility (90-91) | 82.42 | 0.96 | [0.49-1.88] | 1.0000 | 75.82 |  |  |  | 43.96 |  |  |  | 74.44 | 1.00 | [0.56-1.79] | 1.0000 | 31.11 | 0.97 | [0.56-1.67] | 1.0000 |
|  | Health center – by outreach service (313-318) | 69.50 | 0.47 | [0.30-0.74] | **0.0012** | 71.61 |  |  |  | 42.27 |  |  |  | 71.25 | 0.85 | [0.56-1.29] | 0.4629 | 24.13 | 0.68 | [0.45-1.03] | **0.0716** |
| **Health centers grouped acc. to travel time to district hospital** | Provincial hospital (176) | 82.95 | 1.00 |  |  | 81.25 | 1.00 |  |  | 45.45 | 1.00 |  |  | 74.43 | 1.00 |  |  | 31.82 | 1.00 |  |  |
|  | District hospital (175-178) | 84.09 | 1.09 | [0.62-1.91] | 0.8859 | 75.44 | 0.72 | [0.43-1.21] | 0.2446 | 42.13 | 0.87 | [0.57-1.33] | 0.5923 | 72.00 | 0.88 | [0.55-1.42] | 0.6312 | 15.34 | 0.39 | [0.23-0.65] | **0.0004** |
|  | < 30 min (166-168) | 86.90 | 1.36 | [0.75-2.47] | 0.3667 | 80.84 | 0.97 | [0.57-1.67] | 1.0000 | 50.00 | 1.20 | [0.78-1.84] | 0.4485 | 78.92 | 1.29 | [0.78-2.13] | 0.3716 | 24.70 | 0.70 | [0.44-1.13] | **0.1517** |
|  | 30 to 60 min (126-128) | 73.23 | 0.56 | [0.32-0.98] | **0.0463** | 80.47 | 0.95 | [0.53-1.69] | 0.8833 | 47.66 | 1.09 | [0.69-1.72] | 0.7278 | 77.78 | 1.20 | [0.70-2.06] | 0.5859 | 22.83 | 0.63 | [0.38-1.07] | **0.0933** |
|  | > 60 min (111-114) | 50.00 | 0.21 | [0.12-0.35] | **<0.0001** | 51.33 | 0.24 | [0.14-0.41] | **<0.0001** | 26.32 | 0.43 | [0.26-0.71] | **0.0012** | 54.95 | 0.42 | [0.25-0.69] | **0.0008** | 30.36 | 0.93 | [0.56-1.56] | 0.8963 |
| ^1^total numbers for the categories vary between the analyses of the vaccine components; we could not list them individually due to space limitations | | | | | | | | | | | | | | | | | | | | | |
| Variables including seroconversion to individual components that were not significant for at least two of the vaccine antigens are not shown in the table: Number of siblings, Number of household members, Age of mother, Gender of child, Exclusive breastfeeding, Rice in diet, ANC, Tetanus vaccination during pregnancy, Number of Tetanus vaccination during pregnancy and Hepatitis B birth dose | | | | | | | | | | | | | | | | | | | | | |

**Appendix Figure A1** Map of Lao PDR and location of study sites: Vientiane Capital (black circle), Paksan (capital city of Bolikhamxay province, black triangle), Pakkading, Viengthong and Lak Sao (capital cities of Pakkading, Viengthong and Khamkheut district, black square). The travel time by car from Vientiane Capital to Paksan is approximately 3 hours, the travel time from Paksan to either the district capital of Khamkheut or Viengthong is 4-5 hours and the travel time from Paksan to Pakkading is approximately 1 hour. The map was created with QGIS (QGIS Development Team, 2018) using collected GPS‐data and the world borders dataset (http://thematicmapping.org/downloads/world_borders.php, 2019). The data regarding the administrative boundaries of Lao PDR was obtained from the Humanitarian Data Exchange website (https://data.humdata.org/dataset/lao-admin-boundaries, dataset provided by the National Geographic Department of Lao PDR, 2019). Projection used: EPSG 4326 – WGS 84.


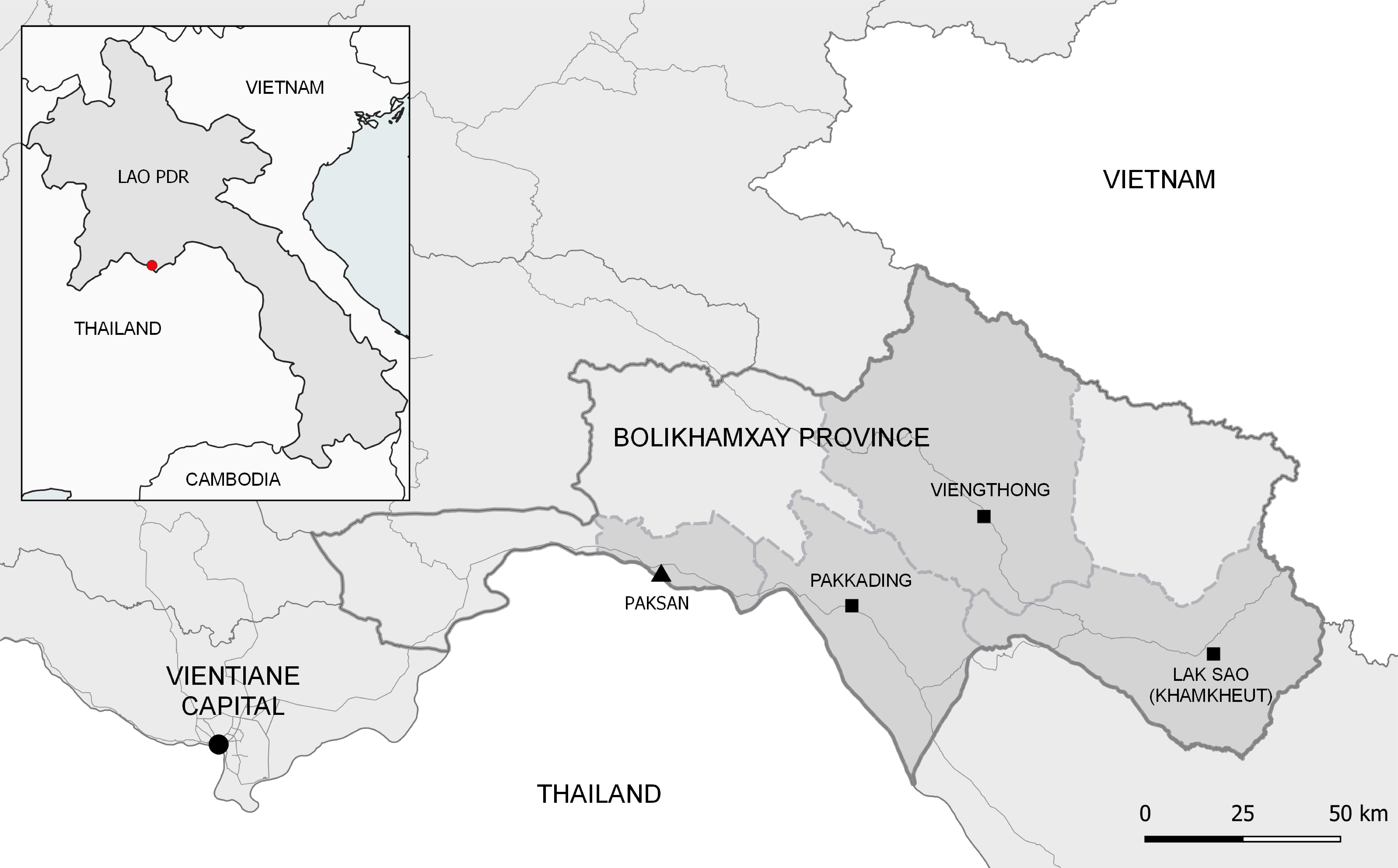


**Appendix Figure A2** Comparison of the serological profiles of children aged 8 to 28 months enrolled in Viengthong district in the same villages in 2017 and 2013/14. Serpoprotection rates are displayed with 95% confidence intervals. ** = p ≤ 0.01, *** = p ≤ 0.001, **** = p ≤ 0.0001.


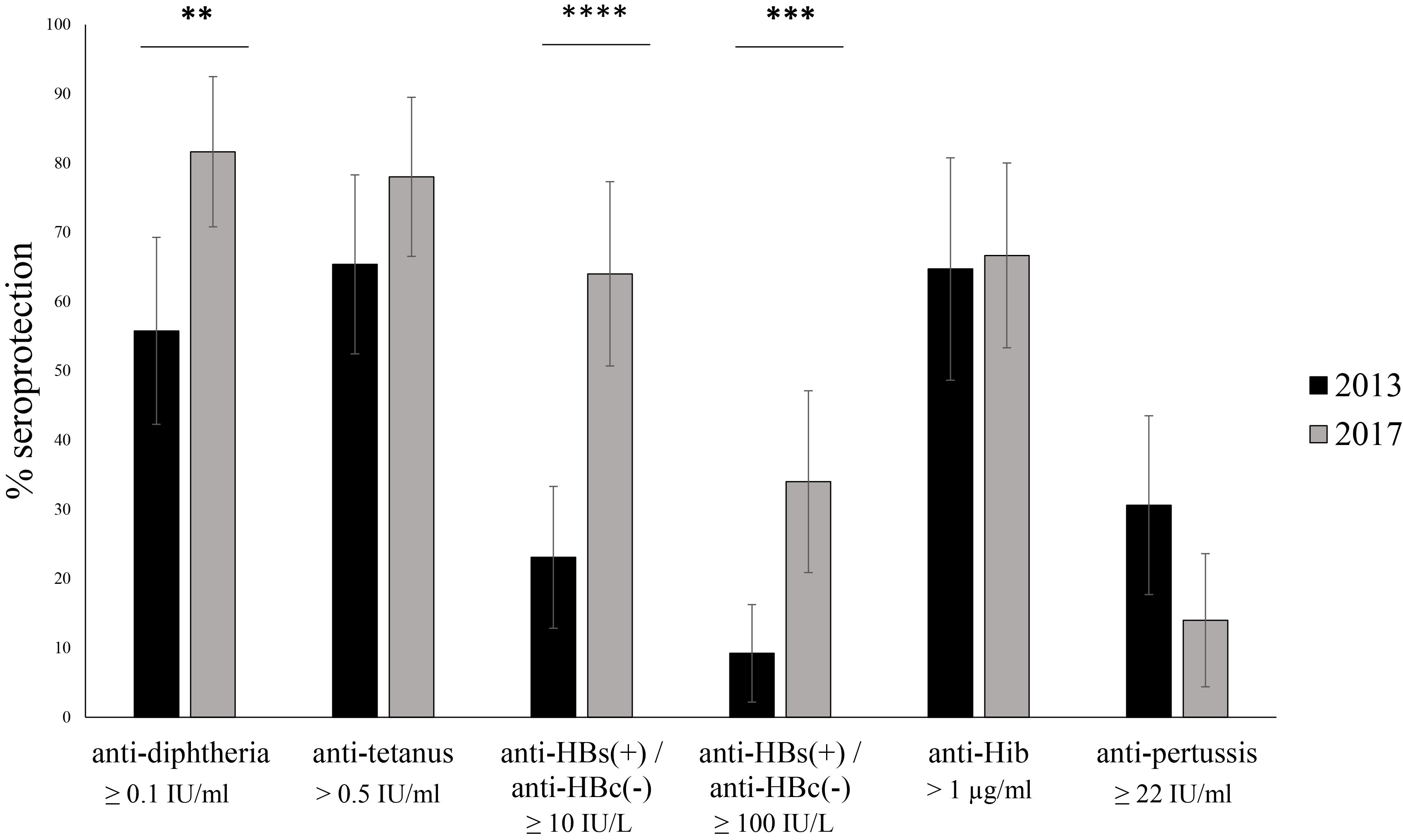

Supplement: ciz143_suppl_Supplementary_Materials [file ciz143_suppl_supplementary_materials.docx]
